# Female sex, poverty and globalization as determinants of obesity among rural South African type 2 diabetics: a cross-sectional study

**DOI:** 10.1186/s12889-015-1622-8

**Published:** 2015-03-27

**Authors:** Oladele Vincent Adeniyi, Benjamin Longo-Mbenza, Daniel Ter Goon

**Affiliations:** Department of Family Medicine, Senior Specialist, Faculty of Health Sciences, Walter Sisulu University, Cecilia Makiwane Hospital, East London Hospital Complex, Private Bag X 9047, Mdantsane, East London South Africa 5200; Department of Community Medicine, Research Champion Professor, Faculty of Health Sciences, Walter Sisulu University, Private Bag X1, Mthatha, South Africa 5117; Department of Nursing Science, Senior Researcher, School of Health Sciences, University of Fort Hare, Private Bag X9083, East London, South Africa 5201

**Keywords:** Type 2 diabetes mellitus, Non-communicable diseases, Obesity, Rural health, South Africa

## Abstract

**Background:**

Countries in Sub-Saharan Africa have recently been experiencing increases in the prevalence of obesity, type 2 diabetes mellitus (T2DM) and other non-communicable diseases in both urban and rural areas. Despite their growing influence on population health in the region, there is a paucity of epidemiological studies on the twin epidemic of obesity and T2DM, particularly in the rural communities in South Africa. We investigated the prevalence and the determinants of overall obesity among patients with T2DM in rural and semi-urban areas surrounding the town of Mthatha, South Africa.

**Methods:**

This hospital-based cross-sectional study was conducted among patients with T2DM attending the outpatient department at Mthatha General Hospital, Eastern Cape Province, South Africa. Data were obtained from 327 participants using standardized questionnaires that included items on sex, age, level of education, type of residence, employment status, smoking status, physical activity, diet and alcohol intake. After taking measurements of height and weight, participants were defined as obese if their body mass index exceeded 30 kg/m^2^. Univariate and multivariate logistic regression analyses were performed to identify the determinants of obesity in our sample population.

**Results:**

We found that 60.2% of our sample population were defined as obese. In our univariate analyses, female sex (p < 0.001), age ≥50 years (p = 0.023), rural residence (p < 0.001), excessive alcohol intake (p = 0.002), current cigarette smoking (p < 0.001), level of education (p < 0.001), regular consumption of soft drinks (p < 0.001) and unemployment (p = 0.043) were found to be positively and significantly associated with obesity. In the multivariate logistic regression analysis, female sex (p < 0.001), unemployment (p = 0.012) and level of education (p < 0.001) were found to be independent determinants of obesity.

**Conclusion:**

We found that female sex, educational attainment, unemployment and current cigarette smoking were positively associated with obesity among the study participants. Lifestyle changes, poverty reduction and public education are urgently needed to address the growing obesity epidemic in rural areas of South Africa.

## Background

The prevalence of overall obesity, defined by body mass index (BMI) of ≥30 kg/m^2^ [1], has reached epidemic proportions worldwide—including in Sub-Saharan Africa [2-10]. Globally, over one billion people are overweight and close to half a billion adults are obese [3]. Although the obesity epidemic was previously thought to be limited to industrialized countries, recent data have emerged showing a substantial increase in prevalence in low and middle-income countries [2,4,11-14]. This trend is mirrored by increases in the prevalence of non-communicable diseases such as diabetes mellitus type 2 (T2DM), hypertension, several types of cancer, gastroesophageal reflux and dyslipidaemia [2,15-21]. Obesity is associated with insulin resistance [20,22-24] and has important implications regarding the aetiology [25,26] and control of T2DM [21]. At the same time, while both obesity and T2DM are associated with an increased risk of mortality due to cardiovascular diseases [13,15-19,21], obesity itself is one of the most significant modifiable risk factors for T2DM [12,21,22,27].

Previous work has identified population ageing, globalization, industrialization, uncontrolled urbanization and the economic transition taking place in many countries as contributors to the twin epidemic of obesity and T2DM in Sub-Saharan Africa [4-7,12,19,28-35]. These processes have resulted in epidemiologic, demographic and nutritional transitions in many countries, which are reflected in population-level changes in diet, physical activity, smoking prevalence and alcohol intake. Together, these factors only exacerbate the challenges posed by diabetes mellitus to health systems [5,6,12,17,19,30,36-45].

While T2DM has been given significant priority by health authorities in South Africa, intervention strategies for the prevention and control of obesity and T2DM have so far been highly fragmented. In addition, little attention has been given to co-morbid patients with obesity and T2DM in rural communities of South Africa who often face challenges in accessing appropriate healthcare [2]. In addition, as reported by Dinsa et al. [46], the association between socioeconomic status and obesity in low income settings is often complex, with a mixed association among men and a negative association among women.

A number of studies on attitudes towards weight and body image in African settings, including those of Holdsworth et al. [47] in Senegal and Kasiam et al. [48] in the Democratic Republic of Congo, have reported that while respondents considered overweight to be desirable, obesity was not considered so. In some African regions, however, obesity is still considered a marker of wealth, success and good health [17,19,36]. Data collected from rural communities are therefore likely to prove valuable for informing intervention programmes, policy development and innovations aimed at addressing inequalities in delivery and access to healthcare. The present study therefore sought to determine the prevalence and determinants of obesity among patients with T2DM in predominantly rural communities surrounding Mthatha, South Africa.

## Methods

### Participants

The study population was drawn from participants of the Diabetes Study in Rural South Africa, a cross-sectional study designed to determine levels of glycaemic control and its determinants among patients with T2DM attending the outpatient department of Mthatha General Hospital, Eastern Cape, South Africa from July to November 2013. The hospital, which forms part of the Nelson Mandela Hospital Complex and acts as a teaching hospital for Walter Sisulu University, is located in the semi-urban township of Mthatha and serves as a referral unit for more than 15 community health centres and clinics in the surrounding rural communities.

Ethical approval was obtained from the ethics committees of Walter Sisulu University, the Eastern Cape Provincial Department of Health and Nelson Mandela Academic Hospital. Each participant provided written informed consent for their participation in the study.

### Sample size

The appropriate sample size was estimated using the following formula:

$$ \mathrm{n} = {\left({\mathrm{Z}}_1-\alpha \right)}^2*\ \left(\mathrm{P}\ \left(1-\mathrm{P}\right)\right)/{\mathrm{D}}^2, $$

where Z is the confidence level, P is the expected proportion of patients with uncontrolled T2DM, and D is the margin of error. P was set at 0.70 and D at 0.05. The calculation was performed at the 95% confidence level. After increasing the final result by 10% to compensate for incomplete laboratory results [49], the required sample size was estimated at 360 participants.

### Eligibility criteria

Participants were eligible for inclusion if they were aged ≥30 years at first diagnosis of diabetes, had received treatment for diabetes for at least one year and were undergoing follow-up care at Mthatha General Hospital. Patients diagnosed with type 1 diabetes and those with a recent diagnosis of diabetes or suffering an acute illness were excluded from the study.

### Sampling procedure

Eligible participants (n = 360) were recruited in series at the hospital outpatient department. A total of 33 were excluded because of incomplete data, resulting in a final sample size of 327.

### Data collection

Participants were interviewed using standardized questionnaires relating to demographic characteristics and lifestyle factors. The questionnaire included items on sex, age, marital status, type of residence, personal monthly income, level of education, employment status, smoking status, alcohol intake, soft drink consumption, diet and physical activity. Data on type 2 diabetes mellitus, receipt of hypoglycaemic agents and insulin therapy, and participation in lifestyle counselling were obtained from patients’ medical records.

The questionnaire was piloted on a group of 20 diabetic patients who were not included in the study sample to ascertain the validity of the instrument. Participants were weighed without heavy clothing to the nearest 0.1 kg using a digital scale (Tanita-HD 309, Creative Health Products, MI, USA). Height was measured to the nearest 0.1 cm using a mounted stadiometer. BMI was then calculated as the ratio of weight in kilogrammes (kg) to height in metres squared (m^2^). Patients whose BMI was greater than or equal to 30.0 kg/m^2^ were defined as obese. These were then categorized according to WHO criteria [50] into class 1 (30.0–34.9 kg/m^2^), class 2 (35.0–39.9 kg/m^2^) and class 3 (≥40.0 kg/m^2^). All other patients were classified as overweight (25.0–29.9 kg/m^2^), normal (18.5–24.9 kg/m^2^) or underweight (<18.5 kg/m^2^).

The socioeconomic factors were measured as a composite of income, level of education and employment status. Monthly income was categorized as low if it was less than 1,200 South African rand (<R1,200) and high if it was greater than or equal to 1,200 South African rand (≥R1,200). Level of education was determined according to the grade level attained in school and participants were categorized as having no formal education, primary (grades 1–6), secondary (grades 7–12) or tertiary (post-secondary) education. Participants were defined as unemployed if they had no occupation in either the formal or informal sector. We hypothesized that these socioeconomic factors would be significantly associated with their obesity status, as shown in previous studies [19,37,38,42,43].

### Lifestyle measures

Data on lifestyle factors such as diet, physical activity, smoking status, and alcohol intake were based on self-reporting. Participants were questioned on their intake of staple foods, fried foods, fast food, fruit, vegetables and red meat. Participants were defined as regular consumers of soft drinks if they consumed these beverages at least twice per day. The categories for smoking status included current smoker (defined as smoking at least one cigarette within the past month), never smoker and former smoker (defined as having quit smoking more than one month prior to the study). Data on current alcohol consumption was considered based on self-reporting, and participants’ intake was categorized according to the guidelines of the Society of Endocrinology, Metabolism and Diabetes of South Africa, which recommend a maximum of two standard units of alcohol per day for men and one for women. Participants were categorized as: never drinkers, excessive drinkers (≥3 units/day for men and ≥2 units/day for women) or moderate drinkers (≤2 units/day for men and ≤1 unit/day for women) [50]. Our measure of physical activity was based on self-reporting and participants were categorized as either inactive or active if they reported engaging in moderate or vigorous exercise leading to an increase in heart rate and respiratory frequency such as gardening and reported fewer than eight hours of television viewing daily.

### Dietary assessment

We obtained 24-h dietary recall data from each participant. The participants were asked how many servings of red meat, fruits, and vegetables they had consumed and how many times they had added salt to their meals. Responses for each item were scored from 1 (one or fewer) to 5 (five or more) portions. Participants were considered to have a high consumption of Western-style fast food (typically including fried chips, red meat and added salt) if they reported eating this type of food ≥4 times/week at fast food restaurants.

### Data analysis

All statistical analyses were performed using the Statistical Package for Social Science (SPSS) version 21 for Windows (SPSS Inc., Chicago, IL, USA). Data were expressed as mean values ± standard deviations (SD) for continuous variables. Frequencies and proportions were reported for categorical variables. Percentages were compared using the chi-square test. Student’s *t*-test was used to compare means between groups. We calculated univariate odds ratios (ORs) using the Maentel–Haenszel test and multivariate ORs and their 95% confidence intervals (95% CIs) using logistic regression analysis to identify the determinants of obesity in our sample. Our logistic regression analysis adjusted for age, type of residence, physical activity, monthly income, soft drink consumption, marital status and alcohol intake). P-values of <0.05 were considered statistically significant.

## Results

Of the 327 study participants, 70.3% were women and 29.7% were men. Participants were typically ≥50 years of age (75.8%), residing in a rural area (88.7%), married (78.3%), unemployed (82.9%), and had at least secondary education (65.7%). All participants reported consuming common staple foods such as pap and umngqusho (corn meal), porridge, meat, rice, bread and potatoes.

**Figure 1:**
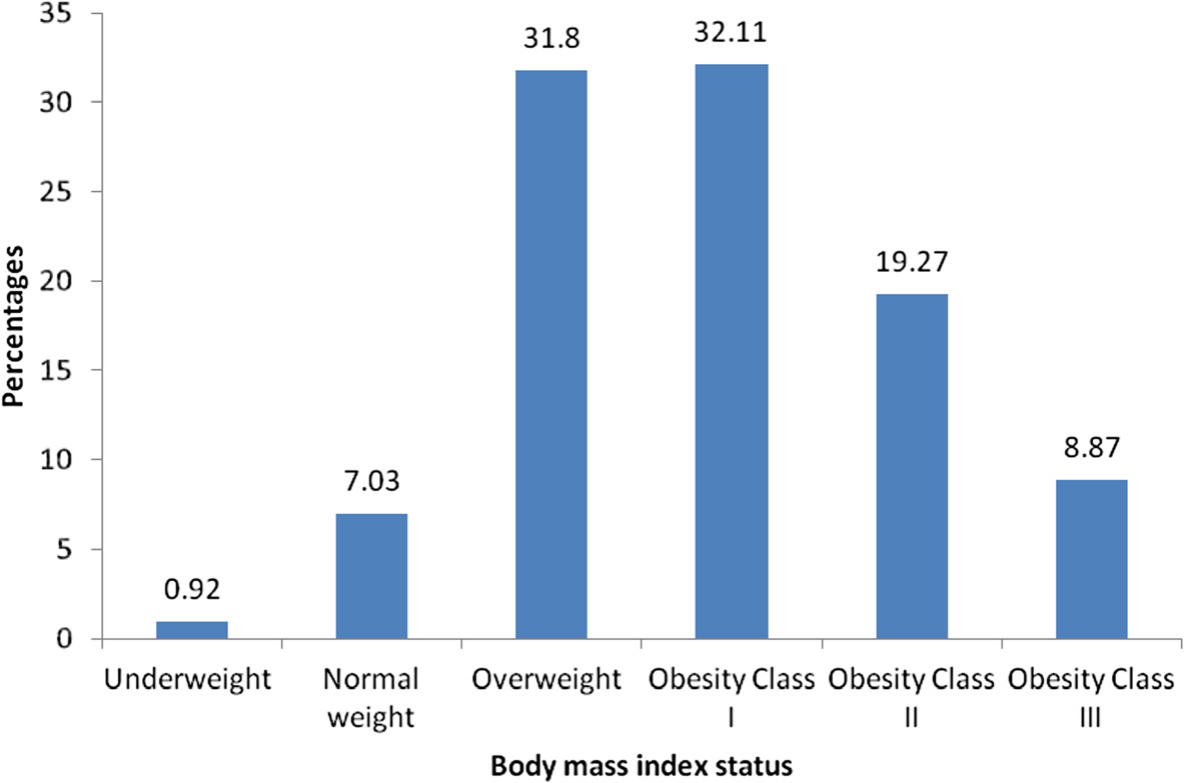
**Distribution of participants according to body mass index status.**

The prevalence of overweight and obesity were 31.8% and 60.2%, respectively. When the obese patients were further sub-categorized, 32.1% were found to have class 1 obesity, 19.3% class 2 obesity and 8.9% class 3 obesity, as shown in Figure 1. Table 1 presents the univariate associations between obesity and female sex, age, marital status, unemployment and soft drink consumption. Our univariate analyses showed that obese patients were significantly more likely to be women (67%, OR: 26.0, 95% CI: 1.6–4.1, p < 0.001), ≥50 years of age (63.7%, OR: 1.8, 95% CI: 1.1–3.0, p = 0.023), single (70.4%, OR: 1.8, 95% CI: 1.0–3.1, p = 0.048), unemployed (62.7%, OR: 1.8, 95% CI: 1.01–3.2, p = 0.043), to have received secondary level (65.8%) or university education (73.7%) and regularly consume soft drinks (67.9%, n = 144/212, OR: 2.5, 95% CI: 1.6–4.0, p < 0.001). The likelihood of becoming obese was nine-fold for physically inactive patients (80.7%, versus 32.9% among physically active participants), four-fold for those with a higher monthly income (70.1%, versus 35.5%) and double for those residing in rural areas (63.6%).

**Table 1:** **Univariate factors associated with obesity**

	**Prevalence of obesity**		
**Variables**	**N (%)**	**OR (95% CI)**	**p-value**
Gender			
Female	154 (67)	26.0 (1.6–4.1)	<0.001
Male	43 (44.3)	1	
Age			
≥50 years	158 (63.7)	1.8 (1.1–3.0)	0.023
<50 years	39 (49.4)	1	
Marital status			
Single	50 (70.4)	1.8 (1.0–3.1	0.048
Married	147 (57.4)	1	
Employment status			
Unemployed	170 (62.7)	1.8 (1.0–3.2)	0.043
Employed	27 (48.2)	1	
Soft drink consumption			
Regular	144 (67.9)	2.5 (1.6–4.0)	<0.001
Irregular	53 (46.1)	1	

The prevalence of obesity among the 321 participants who reported no vegetable intake and regular consumption of Western-style fast food was 98.2%. Table 2 shows the rates of obesity by level of education, alcohol intake, and smoking status. In addition to finding a significant positive gradient (p < 0.001) for the association between obesity and level of education, there were positive associations between alcohol intake and obesity (p = 0.002) and current smoking and obesity (p < 0.001).

**Table 2:** **Prevalence of obesity by level of education, alcohol consumption amount and smoking status among T2DM participants**

**Variables**	**N (%)**
Level of education	
None	17 (37.8)
Primary	37 (55.2)
Secondary	129 (65.8)
Tertiary	14 (73.7)
p-value	<0.001
Alcohol intake	
None	141 (66.8)
Moderate	31 (43.7)
Excessive	25 (55.6)
p-value	0.002
Smoking status	
Never smoker	126 (56.8)
Former smoker	20 (47.6)
Current smoker	51 (81)
p-value	<0.001

The results of our multivariate logistic regression analysis showed that, after adjusting for age, type of residence, physical activity, monthly income, soft drink consumption, marital status and alcohol intake, the most significant determinants of obesity were secondary or tertiary level education groups, current smoking, female sex and unemployment (Table 3).

**Table 3:** **Independent determinants of obesity (Model 1)**

**Variables**	**B**	**SE**	**Wald**	**OR(95% CI)**	**p-value**
Level of education					
Tertiary	1.828	0.672	7.399	6.2 (1.7–23.2)	0.007
Secondary	1.304	0.367	12.648	3.7 (1.8–7.6)	<0.001
Primary	0.657	0.417	2.5	1.9 (0.9–4.4)	0.115
Illiterate			Reference 1		
Smoking history					
Current smoker	1.262	0.473	7.115	3.5 (1.4–8.9)	0.008
Never smoked	0.037	0.368	0.010	1.04 (0.5–2.1)	0.920
Former smoker			Reference 1		
Gender					
Female	0.924	0.267	11.970	2.5 (1.5–4.3)	<0.001
Male			Reference 1		
Employment status					
Unemployed	0.843	0.335	6.330	2.3 (1.2–4.5)	0.012
Employed			Reference 1		
Constant	−2.156	0.555	15.107		<0.001

Adjusted for age, marital status and alcohol intake.

## Discussion

The present study investigated the prevalence and correlates of obesity among patients with T2DM in a rural setting in South Africa. Our results showed that a high proportion of our sample was defined as obese. Our results also confirm the occurrence of epidemiologic and nutritional transitions in these rural communities. Our findings emphasize the urgent need for integrated management approaches to control obesity and T2DM across the country, particularly in underserved rural communities [2]. The South African government has recognized these challenges and has shifted its attention towards addressing the burden of non-communicable diseases, as set out in the National Department of Health 2013–2017 Strategic Plan [27]. While previous studies have highlighted the perceived acceptance of obesity among indigenous African populations [19,36,44], the present study has shed light on the burden of obesity among individuals with T2DM residing in the rural communities of South Africa, and highlights the importance of providing incentives for weight loss and behaviour change in obese individuals with T2DM [51].

According to World Health Organization (WHO), around 600 million adults, or 13% of the global population are currently obese [52], with the prevalence of obesity having doubled between 1980 and 2014. In the same time period, the obesity rate in Sub-Saharan Africa increased from 0.4% to 43% with prevalences for men and women in South Africa reaching 13% and 42%, respectively. Furthermore, 70% of women and 40% of men in South Africa have significantly more body fat than the average for Sub-Saharan Africa [53]. The WHO predicts that the prevalence of type 2 diabetes, whose primary risk factor is obesity, will double across Sub-Saharan Africa in the next 20 years [52].

On the global level, high adult prevalences of co-morbid obesity and T2DM have been reported in both developed and developing nations [54,55]. While The National Health and Nutritional Examination Survey of 1999–2002 reported 85.2% of diabetic patients in the USA were overweight or obese [54], evidence from the UK indicates that around 90% of individuals with diabetes are overweight or obese [55].

The prevalence of obesity in our sample population is consistent with that found in other African settings [56-61] (Table 4). Although our population sample had a number of distinctive characteristics, the findings of the present study can be related to those from both rural and urban areas of Sub-Saharan Africa [13,15-19,21] given that the drivers of the obesity epidemic are similar across these diverse contexts. Our findings demonstrate that widespread obesity is no longer restricted to industrialized countries, given that populations in low and middle-income countries such as South Africa are also significantly affected [4-7,12,32,33].

**Table 4:** **Comparison of the prevalence of diabetes in the sample population and those of other studies**

**Setting**	**Study and sample**	**Proportion of T2DM**	**Obesity**	**Author(s)**
Nigeria, Ekiti North	Cross-sectional; 835 (Semi-urban)	6.8	8.5	Oluyombo et al. [56]
South Africa, Tshwane	Cluster randomized trial; 599 (Urban/rural)	70.3	M = 27; F = 60	Webb et al. [57]
North Africans residing in France	Cross-sectional; 3894 (Urban/rural)	14.0	20.5	Fosse-Edorh et al. [58]
South Africa, Soweto	Longitudinal; 257 (Semi-urban)	All	M = 75; F = 83	Katz et al. [59]
Senegal, Dakar	Cross-sectional; 600 (Urban)	17.8	16.8	Duboz et al. [60]
Cameron, Yaounde, Doula, Bamenda, Bafoussam	Cross-sectional; 2120 (Urban)	T = 15.3; M = 13.7; F = 17.0	28.0	Echouffo-Tcheugui et al. [61]
**South Africa, Mthatha**	**Cross-sectional; 327 (Rural)**	**All**	**60.2**	**Present study**

T = Total; M = Males; F = Females.

The increasing consumption of energy-dense food items such as meats and soft drinks and the gradual decline in the consumption of fruit and vegetables is likely to have had a major impact on the health of our sample population. These effects have been reported in a number of different African settings in previous studies [7,12,19,30,31,34,35]. This is likely to be a result of globalization and the rapid adoption of Western dietary habits, as evidenced by the expansion of fast food restaurant chains in previously underserved rural and semi-urban communities in South Africa and the rest of Sub-Saharan Africa. As such, adopting an individualized approach to patients with co-morbid obesity and T2DM is integral to promoting effective weight loss and achieving glycaemic control.

The over-representation of women in our study population is likely a function of their more effective use of health facilities [45]. More work needs to be conducted into health-seeking behaviour among men, to gain a fuller understanding of the association between obesity and cardiovascular diseases in the wider population. Furthermore, the high proportion of our sample residing in rural areas (88.7%) and experiencing unemployment (82.9%) highlights the role of poverty as a major impediment to attaining good health, both in our sample population and in other African settings [19,44].

The high unemployment rate among our sample may be a function of the high average age and the scarcity of job opportunities in rural South Africa. The majority of the participants earned over R1200 (USD110) per month because of social grants provided by the government. In common with the findings of previous studies [5,6,12,19], the high prevalence of physical inactivity among obese T2DM patients in our study population can be explained by a lack of workplace physical activity, poor access to organized exercise programmes, and the relatively advanced age of the participants.

Our study identified female sex, age (≥50 years), being unmarried, unemployment, higher educational attainment, regular soft drink consumption and current smoking status as determinants of obesity. These factors could be considered to be the results of population ageing, globalization (higher educational attainment, soft drink consumption and tobacco use) and poverty (unemployment, low income and rural residence). These findings are in agreement with previous studies that point to ageing populations, globalization and economic transitions as the main drivers for the twin epidemic of obesity and T2DM in many African countries [4-7,12,32,33]. Similar to the findings of Dinsa et al. [46], the relatively high level of education observed in our study population could also partly explain changes in dietary patterns and preferences. Individuals with higher levels of education are considered more likely in this context to adopt Western dietary habits than their less-educated peers [43]. Although some of these determinants, such as age and sex, are not modifiable, the majority are both preventable and modifiable [12,21,22,27]. Clinicians and policymakers should therefore focus on these determinants to effectively address the twin epidemic of obesity and T2DM.

### Strengths and limitations

This study is the first to address the twin epidemic of obesity and T2DM in rural areas of Eastern Cape, South Africa, and its findings highlight the importance of integrated strategies for managing obesity and T2DM in this setting. It also emphasizes the need for policymakers to recognize the determinants of obesity when developing individualized counselling programmes for weight loss and for systematic reporting of data on non-communicable diseases to promote effective care planning.

Our study had some limitations. First, men were underrepresented in our sample, preventing us from fully understanding their health-seeking behaviour and identifying their specific needs. Second, we cannot rule out the possibility that the use of self-reporting in our data collection could have led to bias. Third, we were unable to obtain in-depth assessments of certain behavioural and lifestyle factors, for example the frequency of alcohol consumption, intensity of physical activity and the micronutrient content of participants’ diets. Our findings should therefore be interpreted with caution.

Finally, although our results showed that current smokers had a higher risk of obesity, and despite the fact that the increase in smoking prevalence across Africa mirrors the unfolding obesity epidemic, we could not identify a plausible biological or behavioural pathway for this association. Our lack of data on smoking cessation in our sample may be another limitation, given that smokers frequently experience weight gain, and by extension an increased risk of T2DM, when they quit smoking. More detailed information on daily cigarette consumption and quit attempts among current smokers may have provided useful insights into the association between smoking and obesity. The cross-sectional nature of our study also prevented us from identifying any causal associations. Further studies are needed to investigate the implications of our findings in the general population.

## Conclusion

Our results suggest the presence of a twin epidemic of obesity and T2DM in the rural communities surrounding Mthatha, South Africa. We found that female sex, level of education, regular consumption of soft drinks, unemployment and current cigarette smoking were all positively associated with obesity. These findings also highlight the ongoing process of globalization, adoption of Western dietary habits and the epidemiologic transitions taking place in rural communities in South Africa. Given their key role in promoting lifestyle changes, poverty reduction and public health education, all of which are essential for mitigating the negative health impacts of these processes, clinicians should be encouraged to be proactive in identifying determinants of obesity among their patients and in providing weight loss counselling to patients with diabetes. In addition, policymakers should place greater emphasis on developing integrated intervention programmes aimed at managing obesity and T2DM to reduce the incidence and burden of cardiovascular disease.

## Abbreviations

T2DM: Type 2 diabetes mellitus
BMI: Body mass index
